# Numerical and Experimental Investigations of Horizontal Turbulent Particle–Liquid Pipe Flow

**DOI:** 10.1021/acs.iecr.2c02183

**Published:** 2022-08-04

**Authors:** ZhuangJian Yang, Chiya Savari, Mostafa Barigou

**Affiliations:** School of Chemical Engineering, University of Birmingham, Edgbaston, Birmingham B15 2TT, U.K.

## Abstract

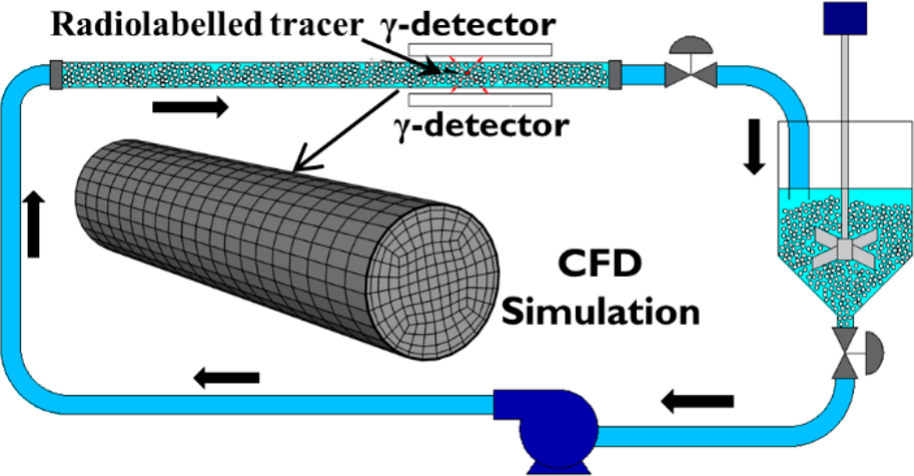

A Eulerian–Eulerian computational fluid dynamics approach is used in conjunction with appropriate auxiliary models for turbulence and solid dynamic properties to study the complex turbulent flow of particle–liquid suspensions in a horizontal pipe. Numerical simulations of the detailed flow field are fully and successfully validated using a unique experimental technique of positron emission particle tracking. The study includes nearly neutrally buoyant as well as dense particles, ranging from small to large at low to high concentrations, conveyed by a Newtonian liquid. Results are analyzed in terms of radial particle and liquid velocity profiles as well as particle distribution in the pipe. The approach provides predictions with a high degree of accuracy. Particle behavior can be classified into three categories depending on their size and particle–liquid density ratio. An analysis of the forces governing the two-phase flow is used to interpret the phenomena observed.

## 1 Introduction

Particle–liquid flow in pipes finds important applications in many industries, including mining, food, energy, chemicals, pharmaceuticals, oil, and construction. Flow geometry, particle characteristics, liquid rheology, and operating conditions have a substantial impact on such a flow. Despite having wide applications, because of their inherent complexity, our ability to design and operate industrial particle–liquid flows is very limited. In consequence, rigorous experimental and numerical investigations are needed to enhance fundamental understanding of the complex multiscale phenomena of hydraulic particle transport in pipes. Imaging of these flows and measurement of their local properties suffer from great difficulties. In practice, particle–liquid flows are usually dense and opaque, making them impossible to visualize by optical laser measurement techniques such as laser doppler velocimetry (LDV) or particle imaging velocimetry (PIV). There have been attempts to study particle–liquid flow in pipes via a number of alternative techniques such as X-ray and electrical resistance tomography.^1,2^ These methods, however, cannot give an accurate pointwise description of the two-phase flow field. These problems can be circumvented by using the unique technique of positron emission particle tracking (PEPT).^3−5^ In this technique, radiolabeled tracers are used to acquire the 3D flow trajectories of the liquid and solid phases in opaque flows and within opaque equipment, with an accuracy comparable to other leading optical methods.^6^

On the computational front, a number of modeling studies have been reported.^7−10^ In computational fluid dynamics (CFD) techniques, the phases in particle–liquid flow can be treated in different ways, that is, Eulerian or Lagrangian. The Eulerian approach utilizes a mesh to describe the fluid domain, while the Lagrangian approach allows particles to move freely in space. The applicability of the Lagrangian approach is usually limited by the number of particles that can be tracked and tends to be computationally expensive even for low concentration flows.^11,12^ On the other hand, the Eulerian–Eulerian method is much more computationally efficient, and its potential has been demonstrated in other flow geometries such as fluidized beds,^13^ stirred vessels,^14^ and viscous pipe flow.^7^ Applications to turbulent particle–liquid pipe flow have been mainly concerned with the flow of very fine particle slurries such as sand with studies of flows conveying larger particles being scant and in general not comprehensively validated.^15−18^ To help accurate prediction of the behavior of particles via the Eulerian–Eulerian approach, the implementation of kinetic theory of granular flow (KTGF) models to estimate solid viscosities and solids stresses has been advocated.^19,20^

In this paper, we use CFD based on the Eulerian–Eulerian numerical approach with appropriate KTGF models to study the turbulent horizontal pipe flow of nearly neutrally buoyant as well as dense particles conveyed by a Newtonian liquid. The numerical model is fully validated by using pointwise experimental measurements of local phase velocity and concentration obtained from the unique PEPT technique. A parametric study is then conducted to elucidate the complex phenomena that govern such flows under various conditions of particle size, density, and concentration. The aim of this work is to evaluate the capability of the numerical approach adopted and demonstrate its potential in aiding the design and operation of such complex processes.

## 2 Experimental Section

### 2.1 Pipe Flow Loop

**Figure 1:**
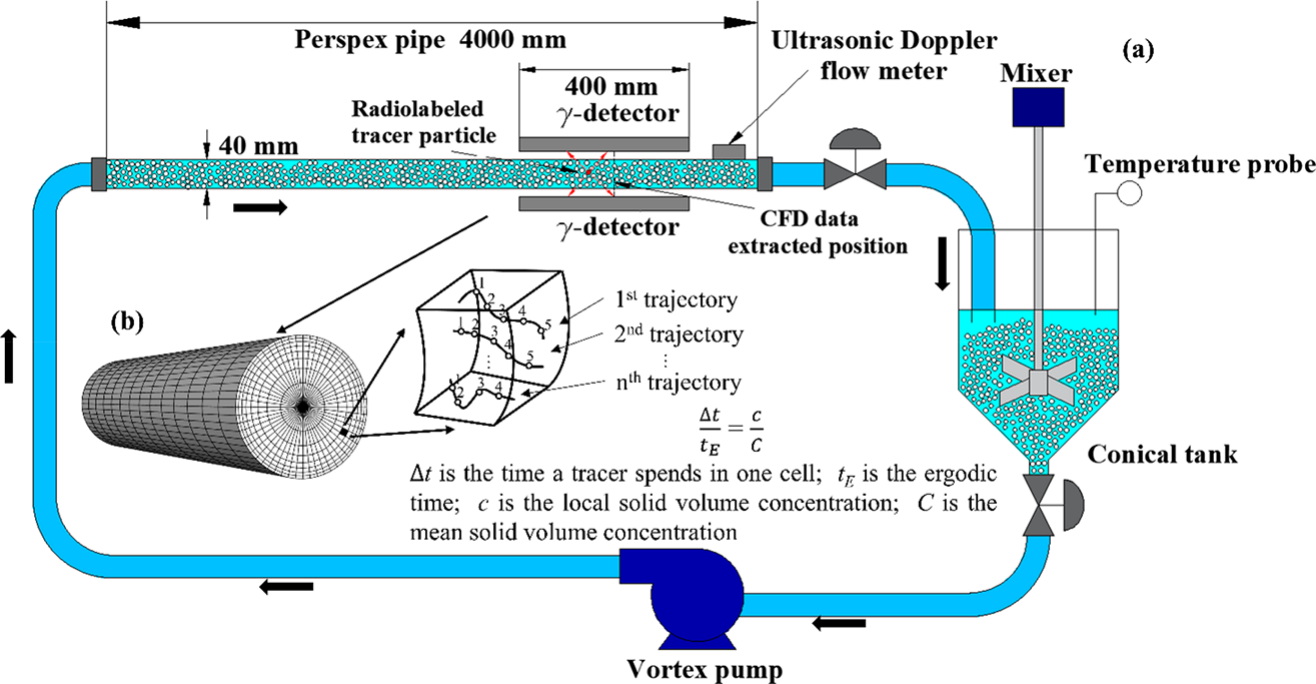
(a) Experimental pipe flow loop and PEPT setup, (b) illustration of cylindrical grid of equal-volume cells used for analysis of Lagrangian PEPT data.

The experimental pipe flow loop used to study the horizontal flow of particle–liquid suspensions is schematically represented in Figure 1a. The two-phase flow was driven by a vortex pump (T21–32 HF4 LB1, Turo vortex pump, EGGER, Switzerland) through a perspex pipe of 40 mm internal diameter. The flow imaging section was 400 mm long, beginning 3 m downstream of the upstream pipe bend to ensure fully developed flow free from bend effects.^21^ The volumetric flow rate of the mixture was measured in situ by a Doppler ultrasonic flow meter (UF D5500, Doppler flow meter, Micronics) and was independently confirmed at the outlet of the pipe by stopwatch and bucket measurements from which the mean particle delivery concentration was deduced. The suspension was circulated until it reached steady-state and a constant temperature before any measurements were taken. The carrier fluid was a 36 wt % aqueous sugar solution of Newtonian rheology. The dispersed phase consisted of monosize Calcium alginate particles of nearly spherical shape, fabricated in-house according to the protocol reported in Fairhurst et al.^3^ Different particle sizes, densities, and concentrations were investigated, as summarized in Table 1. Particle density was controlled by adding silica powder to the alginate solution.

**Table 1:** Properties of Solid Particles and Liquid Phase Used in the Experiments

	nearly neutrally buoyant particles	dense particles
*d*_*p*_ (mm)	2 ± 0.05, 4 ± 0.14	2 ± 0.07, 4 ± 0.18
ρ_*s*_ (kg/m^3^)	1165 ± 3	1248 ± 3
ρ_*L*_ (kg/m^3^)	1143 ± 2	1145 ± 2
ρ_*r*_ (−)	1.02	1.09
*C*_*s*_ (vol %)	6, 12, 21, 31	6, 12, 24, 33
μ_*L*_ (Pa s)	0.0043 ± 0.0003	0.0043 ± 0.0003
*u*_*m*_ (m/s)	0.75 ± 0.02	0.75 ± 0.03

### 2.2 Positron Emission Particle Tracking

PEPT is a nonintrusive measurement technique that uses suitable positron emitting particle tracers to track the components of the flow in three-dimensional space and time, and accurately determine their long-term trajectories. Particle tracking can be achieved in opaque fluids and inside opaque equipment. This is a unique advantage over other leading optical visualization techniques, while, as shown in our previous work, its accuracy is comparable to that of PIV.^6^ PEPT has been extensively used to study a variety of flows. More information about the technique, its hardware, and software has been published in our earlier papers.^22−27^ In a pipe, flow imaging by PEPT usually consists of letting a single particle tracer flow in a closed loop until it maps the whole area of interest, thus requiring a statistically representative number of trajectories, usually >50.^28,29^ In this study, to enhance data statistics and reduce experimental time, several tracers were sequentially introduced in the flow, thus yielding about 500 trajectories in each experiment. Both the solid and liquid phase were individually tracked in separate consecutive experiments. For the liquid, a 400 μm neutrally buoyant resin particle tracer was used. A similar resin particle tracer was encapsulated inside a representative alginate particle and used to track the solid phase.

## 3 PEPT Data Analysis

### 3.1 Radial Velocity Profiles of Liquid and Solid Phases

**Figure 2:**
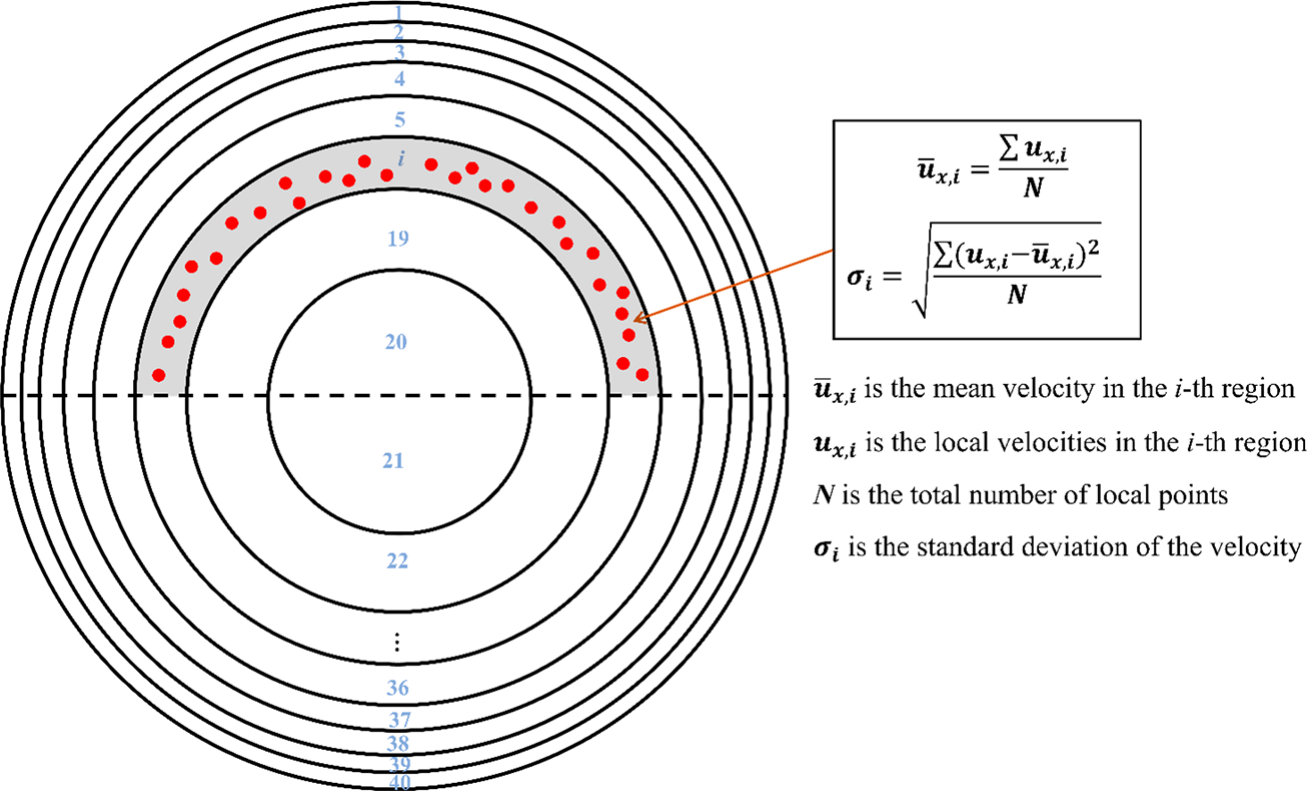
Pipe cross-section divided into 40 semiannular equal volume regions for liquid and solid velocity profile calculations.

As PEPT provides 3D particle tracer locations at various times (Figure 1b), the velocity of both phases at each detected tracer position can be calculated from time derivatives of neighboring positions using the differencing method. In pipe flow, only the axial velocity component (*u*_*x*_) is of importance and radial motion is negligible. The axial velocity can be calculated from the slope of a line fitted to a number of *x*-locations vs time using regression analysis.^28^ Ten consecutive *x*-locations of the particle tracer were used covering a small distance less than 20 mm so that the estimated velocity was truly axial and the effects of any fluctuations in the radial direction were minimized. The local velocity of each phase was obtained by dividing the pipe cross-section into 40 semiannular regions of equal area; thus, the radial velocity profile was constructed. Because of the asymmetric nature of the suspension flow, 20 regions above and 20 regions below the pipe centerline were used, as depicted in Figure 2. The mean and standard deviations of the local velocity in each semiannular region were calculated. The mean mixture velocity, *u*_*m*_, was used to normalize the mean velocity in each region.

### 3.2 Local Particle Concentration

In addition to the radial velocity profile of each phase, the Lagrangian trajectories provided by PEPT were also used to infer the solid phase distribution across the pipe from the local occupancy of the tracer using a 3D measurement grid consisting of 1700 equal volume cells, as shown in Figure 1b. Local occupancy has traditionally been defined as the time a tracer spends in each grid cell during the experiment, divided by the total experimental time (*t*_∞_). This definition, however, is highly dependent on the density of the grid used and, as such, local occupancy tends to zero as the number of cells increases.^23^ This grid-dependence can be circumvented by using the ergodic time (*t*_*E*_), which is the time a tracer would spend inside a cell if the flow were single phase and ergodic. If the cells are chosen to have equal volume, however, the ergodic time can be defined as the total experimental time divided by the total number of cells (*t*_*E*_ = *t*_∞_/*N*_*c*_). The ergodic time assumes that the tracer has an equal probability of visiting any cell in the grid. The local occupancy (*O*_*E*_) can then be expressed as Δ*t*/*t*_*E*_, where Δ*t* is the total cumulative time the tracer spends within a given cell during all its visits (Figure 1b). We showed in our previous work that *O*_*E*_ = *c*/*C*_*s*_, where *c* is the local solid volume concentration and *C*_*s*_ is the mean volume concentration of solids.^23^

## 4 Numerical Modeling

The two-fluid Eulerian–Eulerian model was used to simulate turbulent particle–liquid flow in a horizontal pipe and results were validated against PEPT measurements, as described above. Experiments were conducted at a typical industrial liquid Reynolds number . As the solid phase was denser than the liquid phase, the inhomogeneous model was used. The drag force, lift force, turbulence dispersed force, and virtual mass force were all included in the simulations. Moreover, appropriate KTGF models to predict the solid phase pressure were used.^19^

### 4.1 Conservation of Mass and Momentum

The relevant equations are widely available in the literature.^30^ The mass conservation equation of liquid and solid phases can be expressed as
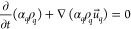
1where *t* is time, α_*q*_ is the volume fraction of the *q*th phase, and ρ_*q*_ and *u⃗*_*q*_ are its density and velocity, respectively.

The momentum conservation equation for the liquid and solid phase is, respectively, given by
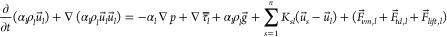
2and,
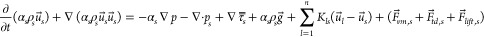
3where *p* is pressure and *g* is gravitational acceleration. *p*_*s*_ is the solid phase pressure estimated by KTGF models. *K*_*ls*_ and *K*_*sl*_ are the momentum exchange coefficients representing interphase forces. *F⃗*_*vm,q*_, *F⃗*_*td,q*_, and *F⃗*_*lift,q*_ are, respectively, the virtual mass force, turbulent dispersion force, and lift force of the *q*th phase, and τ̿_*s*_ is its stress–strain tensor, expressed as
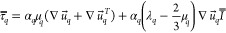
4where μ_*q*_ and λ_*q*_ are the shear viscosity and bulk viscosity. *u⃗*_*q*_^*T*^ indicates the impact of dilation and *I̿* is the unit tensor.

The force models used include the Gidaspow drag force model,^31^ Moraga lift force model,^32^ and Burns et al.’s turbulent dispersion model.^33^ The virtual mass force model is given by Drew and Lahey,^34^ while the turbulence interaction is accounted for by Simonin et al’s model.^35^

### 4.2 Kinetic Theory of Granular Flow

To calculate the solid stress–strain tensor and solid pressure in eqs 3 and 4, the kinetic theory of granular flow method is used, where the solid stress and solid viscosities are a function of the granular temperature. The solid shear viscosity is defined as
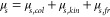
5where μ_*s,col*_, μ_*s,kin*_, and μ_*s,fr*_ are the collisional viscosity, kinetic viscosity, and frictional viscosity, respectively. The collisional viscosity is defined as^36^
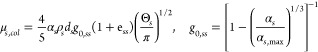
6where α_*s*_ and ρ_*s*_ are the solid volume fraction and density, Θ_*s*_ is the granular temperature, *g*_0,*ss*_ is the radial distribution function of the solid phase, and *e*_*ss*_ is the restitution coefficient for particle collision.

For high solid concentrations (>20 vol %), the kinetic viscosity is usually obtained from^36^
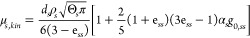
7

For lower solid concentrations, however, the correlation of Syamlal et al.^37^ is used instead
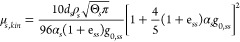
8

The generally adopted values for the friction packing limit of solids and the maximum packing volume fraction are 0.61 and 0.63, respectively. Therefore, the frictional viscosity arising from friction between particles is normally activated when the local solid volume fraction exceeds 0.6, thus^38^
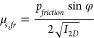
9

The Johnson-Jackson model is used to calculate the frictional pressure^39^
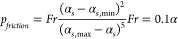
10

The resistance of the compression and expansion of solids are estimated using the modified bulk viscosity, λ_*s*_^40^
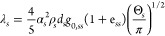
11

For low solid concentrations, the solid pressure is usually expressed as^37^
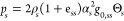
12

For high concentrations, however, the following relationship is usually preferred as it is less prone to divergence^40^
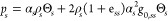
13In conclusion, by introducing the granular temperature into the momentum eq (eq 3), the following transport equation is finally obtained for the solid phase
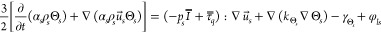
14

For low solid concentrations, the diffusion coefficient is estimated from^37^
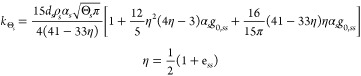
15and for high solid concentrations, it is estimated from^36^
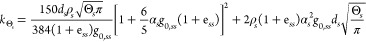
16

In addition, the collisional dissipation energy term is obtained from^40^
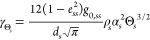
17and the transfer of kinetic energy term is estimated from^36^
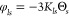
18

### 4.3 Turbulence Model

There are different CFD models to simulate turbulence. Here, we used the shear-stress transport (SST) model as it is generally recognized to be the most suitable for *Re*_*L*_ < 10^4^.^41^ The turbulence kinetic energy for the *q*th phase is expressed as
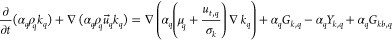
19and the specific turbulence dissipation rate is given by
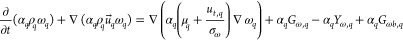
20where *k* is the turbulent kinetic energy and ω is the specific turbulence dissipation rate. *G*_*k,q*_ and *G*_*ω,q*_ are the generation of turbulence kinetic energy and specific dissipation rate. *Y*_*k,q*_ and *Y*_*ω,q*_ are the dissipation of the turbulence kinetic energy and specific dissipation rate. *G*_*kb,q*_ and *G*_*ωb,q*_ are the generation of the turbulence kinetic energy and specific dissipation rate due to buoyancy effects.

### 4.4 Simulation Setup

Simulations were performed in 3D due to the axial asymmetry of the particle–liquid flow, using the commercial ANSYS Fluent 2021R1 platform. The physical geometry, computational mesh, boundary conditions, and solver setup are described below.

#### 4.4.1 Geometry and Mesh

The geometry set up in the simulation was a replica of the experimental flow pipe and was meshed by O-grid hexahedral cells. Since flow is only weakly turbulent (*Re*_*L*_ < 10 000), *y*^+^ was selected to be <15.^42^ A mesh independence study resulted in a mesh growth rate of 1.2, giving approximately 9.6 × 10^5^ cells with a first cell height of about 0.4 mm. The majority of the mesh had an aspect ratio less than 1.6 and a skewness less than 0.2.

#### 4.4.2 Boundary Conditions

The inlet liquid and solid velocities were set values equal to the measured mean mixture velocity, and at the exit the pressure was set to atmospheric. The no-slip condition was used for the liquid at the pipe wall. For particles, a free-slip condition was set by using a value of 0.451 for the specularity coefficient.^17^ The effects of particle-wall collisions were included via a particle-wall restitution coefficient of 0.99.^17^

#### 4.4.3 Solver Setup

The SIMPLE scheme was used for pressure–velocity coupling due to its efficiency and robustness.^17^ For increased accuracy, the second-order scheme was used for both pressure and momentum terms. The pressure, momentum, and volume fraction under-relaxation factors were set to 0.2, 0.3, and 0.4, respectively. The under-relaxation factors for all other parameters were set to their default values in the software. As the particle–liquid flow was steady and fully developed, simulations were run in steady-state mode. The root–mean–square (RMS) residual criterion was set to 10^–4^. A lower criterion led to convergence problems in some cases, especially when dealing with high solid volume fractions.

## 5 Results and Discussion

**Figure 3:**
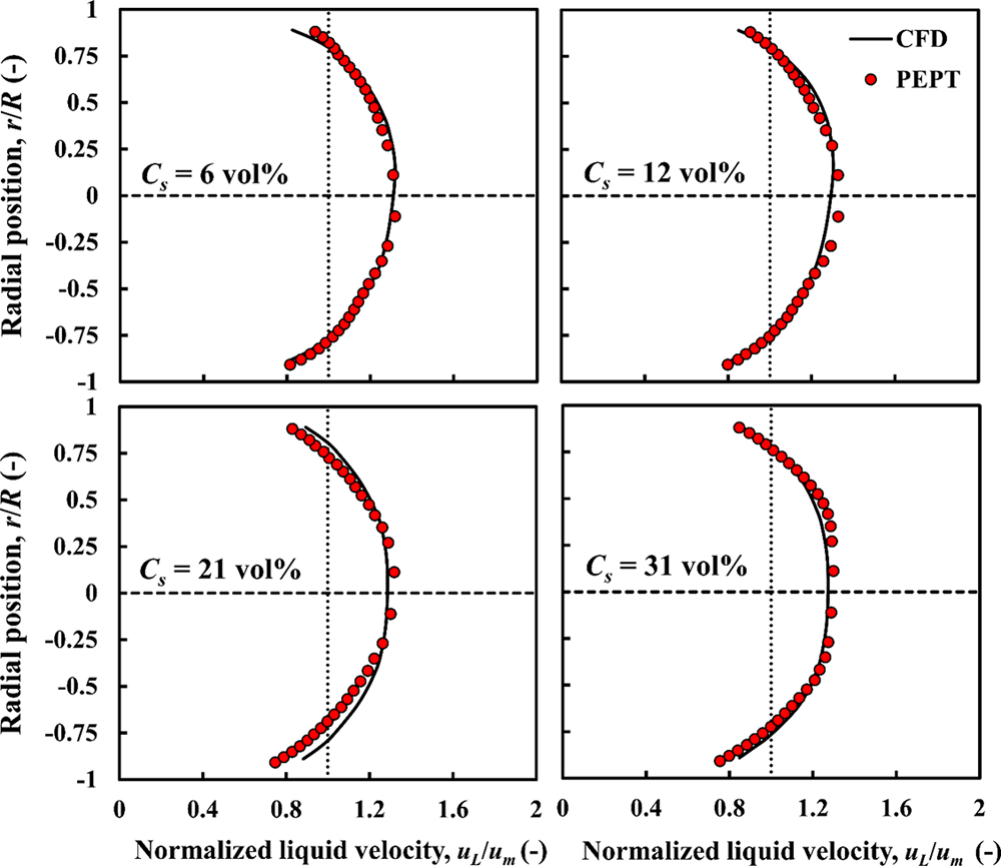
CFD-predicted and experimental PEPT liquid velocity profiles compared: nearly neutrally buoyant particles, ρ_*r*_ = 1.02; *d*_*p*_ = 4 mm.

**Figure 4:**
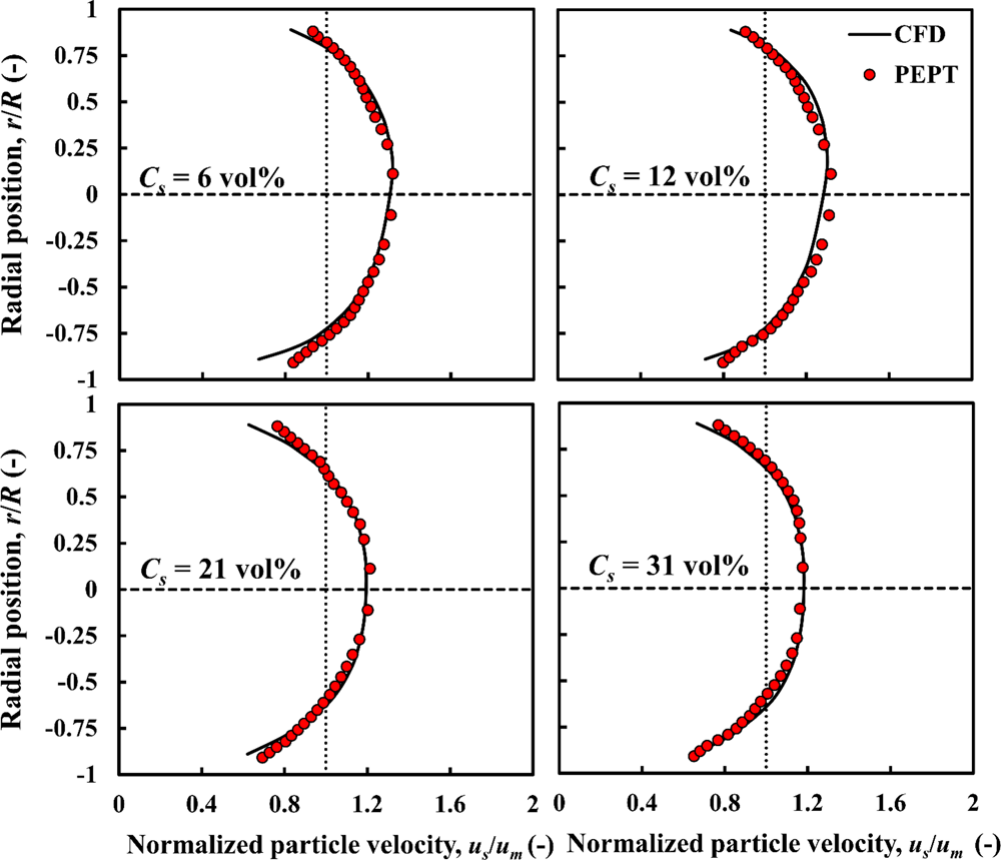
CFD-predicted and experimental PEPT particle velocity profiles compared: nearly neutrally buoyant particles, ρ_*r*_ = 1.02; *d*_*p*_ = 4 mm.

**Figure 5:**
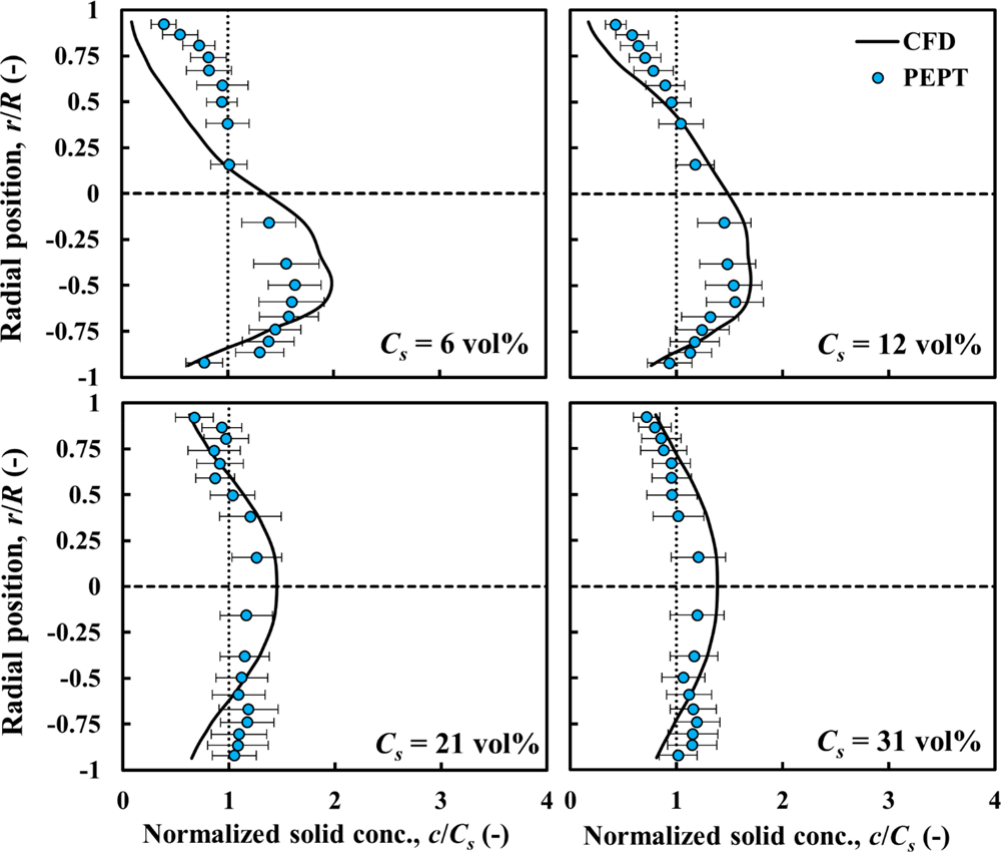
CFD-predicted and experimental PEPT particle concentration profiles compared: nearly neutrally buoyant particles, ρ_*r*_ = 1.02; *d*_*p*_ = 4 mm.

**Figure 6:**
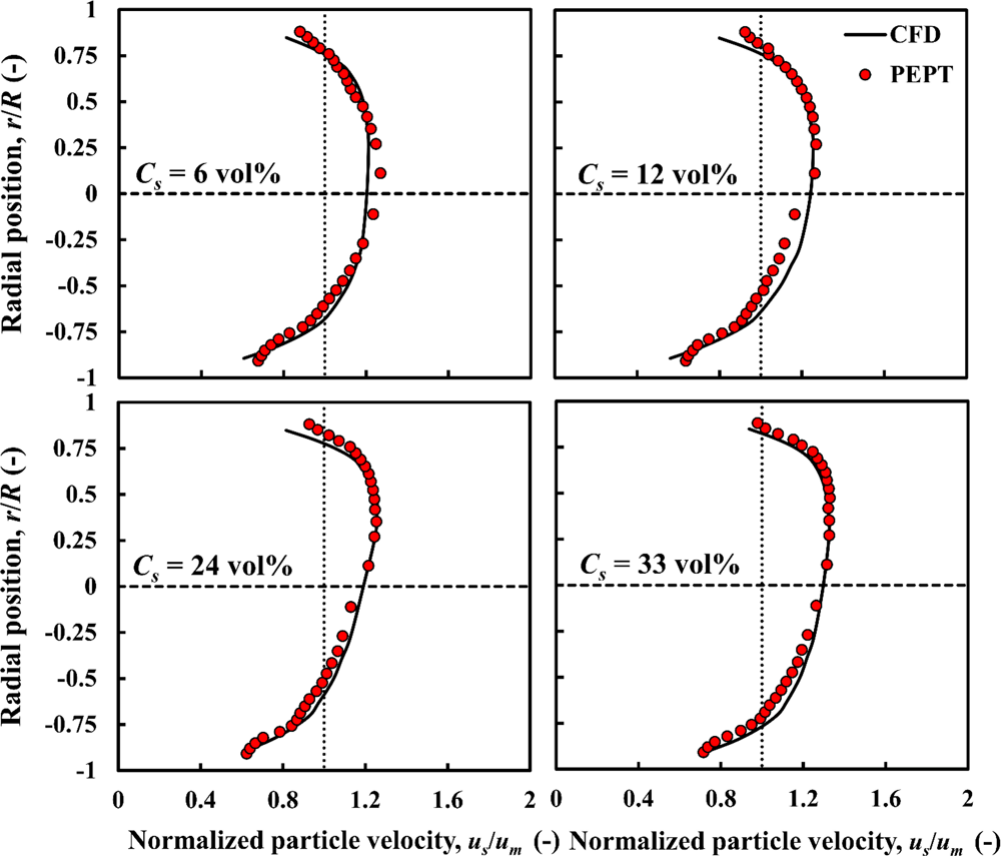
CFD-predicted and experimental PEPT particle velocity profiles compared: dense particles, ρ_*r*_ = 1.09; *d*_*p*_ = 4 mm.

**Figure 7:**
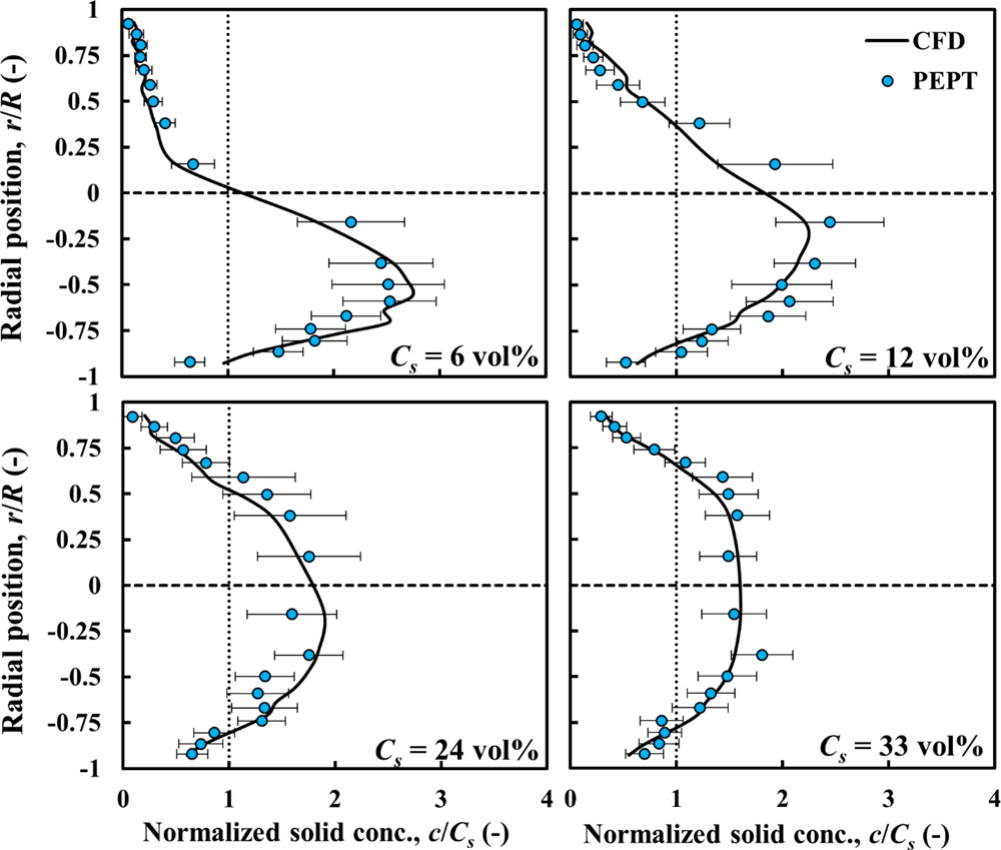
CFD-predicted and experimental PEPT particle concentration profiles compared: dense particles, ρ_*r*_ = 1.09; *d*_*p*_ = 4 mm.

The CFD results were validated by comparing the predicted velocity profiles of the two phases as well as the solid phase distribution with those determined experimentally by PEPT (Figures 3–7). The simulation results were examined at a section where the two-phase flow was fully developed, coinciding with the middle point of the field of view of the PEPT detectors (Figure 1a). While validation results are presented for the case of 4 mm particles, additional validation data for 2 mm particles are included in the Supporting Information.

### 5.1 Model Validation

#### 5.1.1 Nearly Neutrally Buoyant Particles

Figures 3 and 4 show the validation of the liquid and particle velocity distributions for the case of nearly neutrally buoyant particles at different mean solid concentrations. The error bars of the experimental velocity profiles are too small to be shown, and there is excellent agreement between CFD and PEPT at all solid loadings. The radial velocity profiles for both liquid and particles are approximately symmetrical about the centerline. At high concentrations (21, 31 vol %), the liquid and solid velocity profiles exhibit a central faster-moving flat core region, having a velocity ∼1.20*u*_*m*_. The liquid and particles in the surrounding annular region interact with the pipe wall and move with lower velocities. Moreover, the particle velocity profiles match closely the liquid velocity profiles, that is, nearly zero slip velocity, as expected for nearly neutrally buoyant particles.

The particle distribution profiles corresponding to the above cases are presented in Figure 5. The radial particle distribution is obtained by considering 40 pipe sections spanning the field of view (400 mm) of the PEPT camera, where the flow is fully developed. An overall radial average profile with its corresponding standard deviation is estimated by constructing the local particle concentration in each pipe section and then averaging over the 40 sections. There is close agreement between the CFD and experimental profiles, with most of the predicted profile, in each case, being within the experimental error bars. At the lowest solid concentration (*C*_*s*_ = 6 vol %), most of the particles tend to show significant accumulation at the bottom part of the pipe due to gravitational effects. At *C*_*s*_ = 12 vol %, the trend is similar but with more particles moving in the upper part of the pipe cross-section, thus shifting the maximum slightly higher toward the center. At the higher concentration of *C*_*s*_ = 21 vol %, the solid distribution volume is approximately symmetrical about the centerline; the maximum in the faster-moving core region being ∼1.4*C*_*s*_. At the highest concentration of *C*_*s*_ = 31 vol %, the distribution retains it symmetry but becomes flatter as the maximum decreases to ∼1.2*C*_*s*_. This indicates that concentrated flows of nearly neutrally buoyant particles tend to flow in the homogeneous flow regime as particle–particle interactions overcome gravitational effects.

#### 5.1.2 Heavier Particles

The particle–fluid density ratio (ρ_*r*_) plays an important role. To investigate the effects of increasing ρ_*r*_ to 1.09, the density of the particles was adjusted by adding silica powder to the alginate solution while keeping other particle properties unchanged. The CFD-predicted and experimental velocity profiles of the solid phase are compared at different mean solid concentrations in Figure 6, where a very good agreement is observed. For *C*_*s*_ = 6 and 12 vol %, the velocity profiles are slightly asymmetrical, with a maximum located well above the centerline. The degree of asymmetry increases further at the higher concentrations used (*C*_*s*_ = 24 and 33 vol %). Compared to the nearly neutrally buoyant particles, the asymmetry is much more pronounced due to increased gravitational settling.

Validation results for the solid phase distribution are depicted in Figure 7, showing all CFD predictions within the error bars of the experimental results. At *C*_*s*_ = 6 and 12 vol %, particles tend to accumulate at the bottom of the pipe, similar to the nearly neutrally buoyant particles. However, the solid distribution profiles are much sharper, with a maximum reaching 2.8*C*_*s*_ at 6 vol % and 2.3*C*_*s*_ at 12 vol %. Again because of enhanced particle–particle interaction, flow asymmetry reduces as solid concentration increases, with particle distribution becoming almost symmetrical at *C*_*s*_ = 33 vol %, with a maximum at the center.

In conclusion, the above validation has shown that the Eulerian–Eulerian approach with appropriate KTGF models is robust and is capable of giving reliable predictions of phase velocity as well as spatial particle distribution in turbulent pipe flow of nearly neutrally buoyant as well as dense particles. The capabilities of this successful approach will now be exploited to conduct a detailed parametric study to unravel the effects of the various parameters that govern particle–liquid flow and, in particular, the phenomena that characterize particle behavior.

### 5.2 Parametric Study

Simulations were performed for different particles sizes (*d*_*p*_ = 0.1–10 mm, i.e., *d*_*p*_/*D* = 0.0025–0.25) and different particle densities (ρ_*r*_ = 1.02, 1.09, 1.5), at a mean particle concentration *C*_*s*_ = 30 vol %, and for the same mixture flow rate used in the above validation study. Results are presented and discussed in terms of liquid/particle velocity and solid concentration profile plots. The models used to estimate the relevant forces (virtual mass, turbulence dispersion, lift, drag, turbulence interaction) are the same throughout and are not subject to variation as a function of flow conditions. The effects of varying particle size and density in the parametric study are taken care of by the KTGF model.

#### 5.2.1 Effects of Particle Size and Density

**Figure 8:**
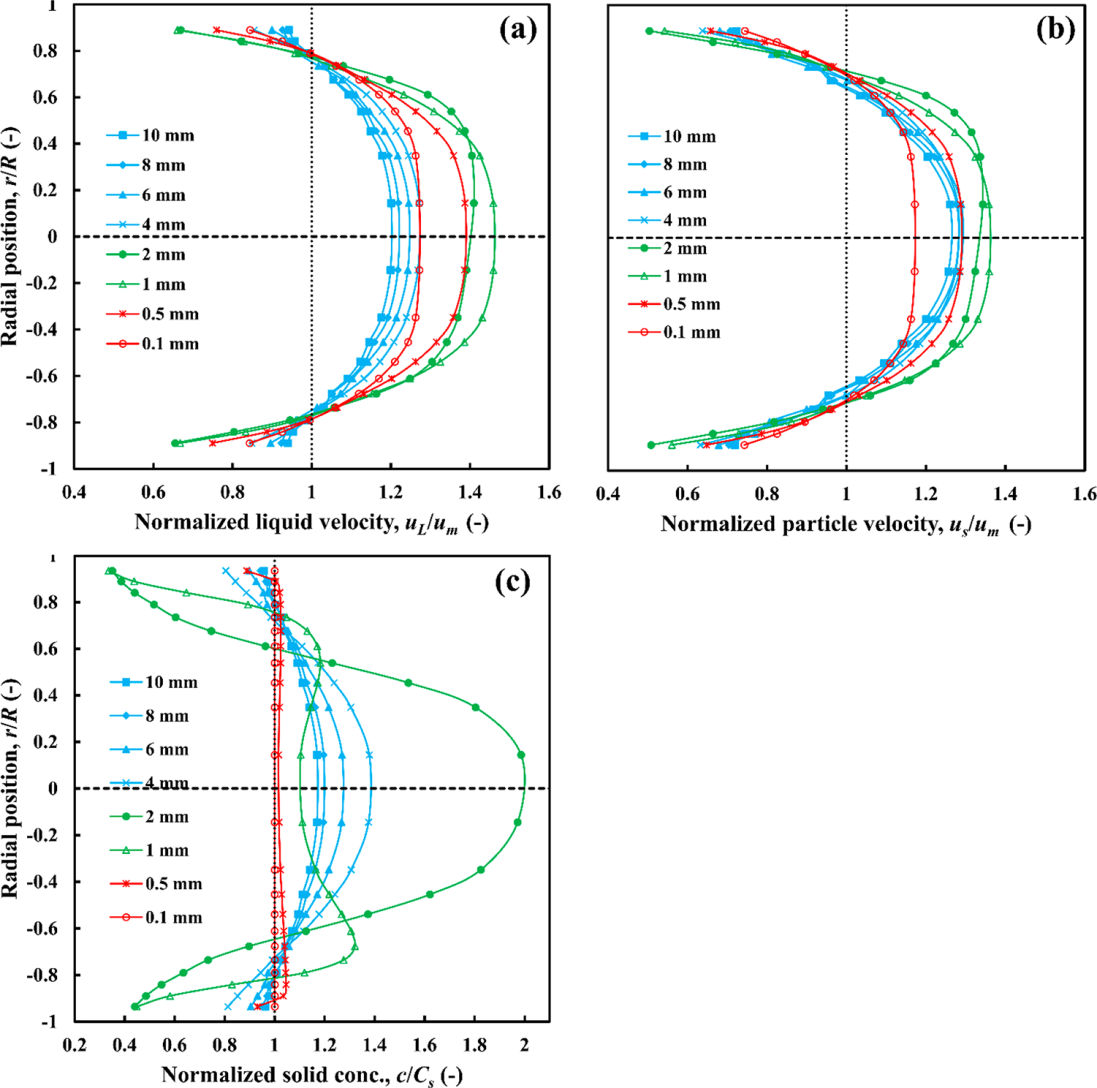
Effects of particle size on radial profiles of (a) liquid velocity, (b) particle velocity, and (c) particle concentration, *C*_*s*_ = 30 vol %, ρ_*r*_ = 1.02.

**Figure 9:**
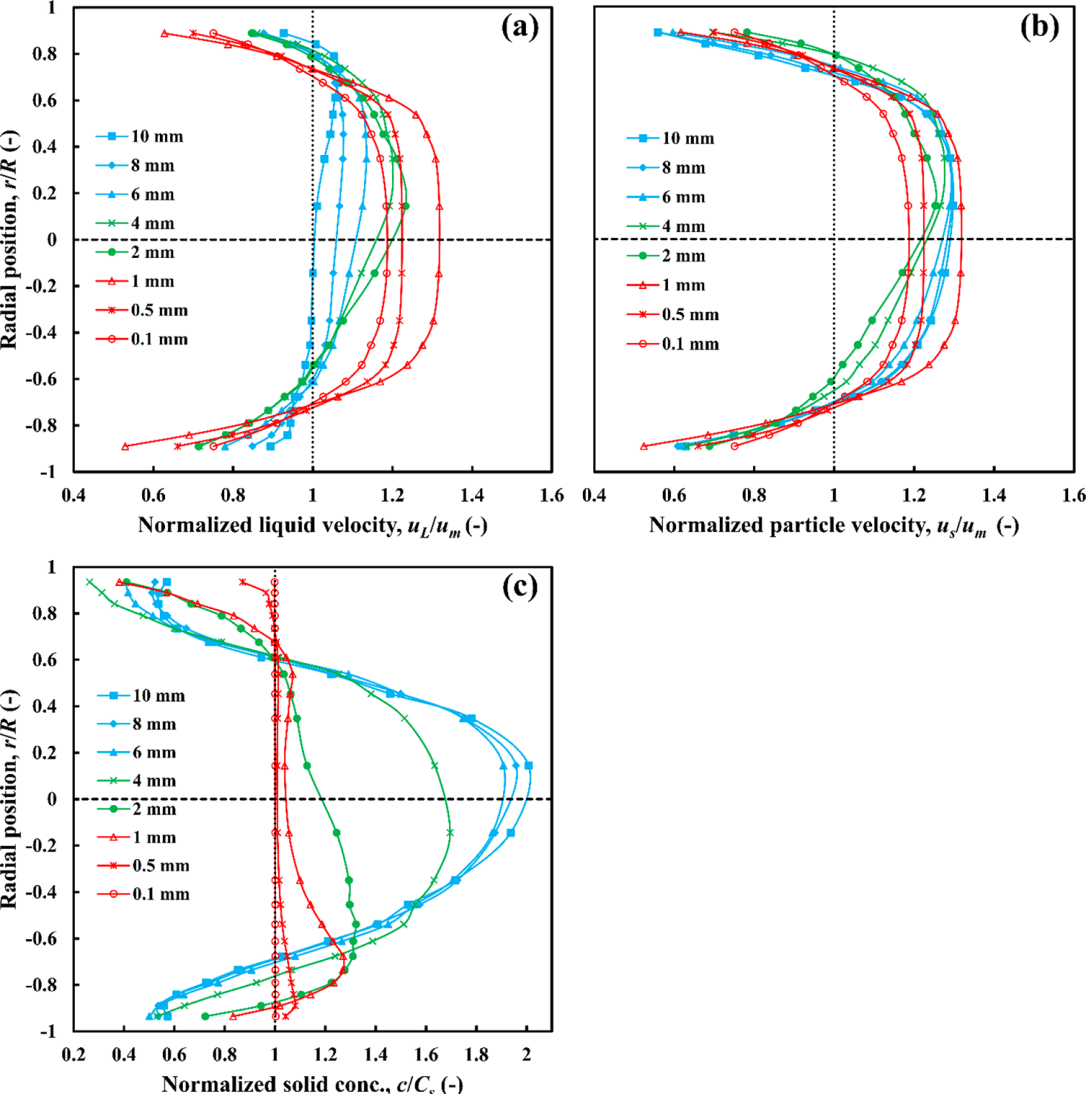
Effects of particle size on radial profiles of (a) liquid velocity, (b) particle velocity, and (c) particle concentration, *C*_*s*_ = 30 vol %, ρ_*r*_ = 1.09.

**Figure 10:**
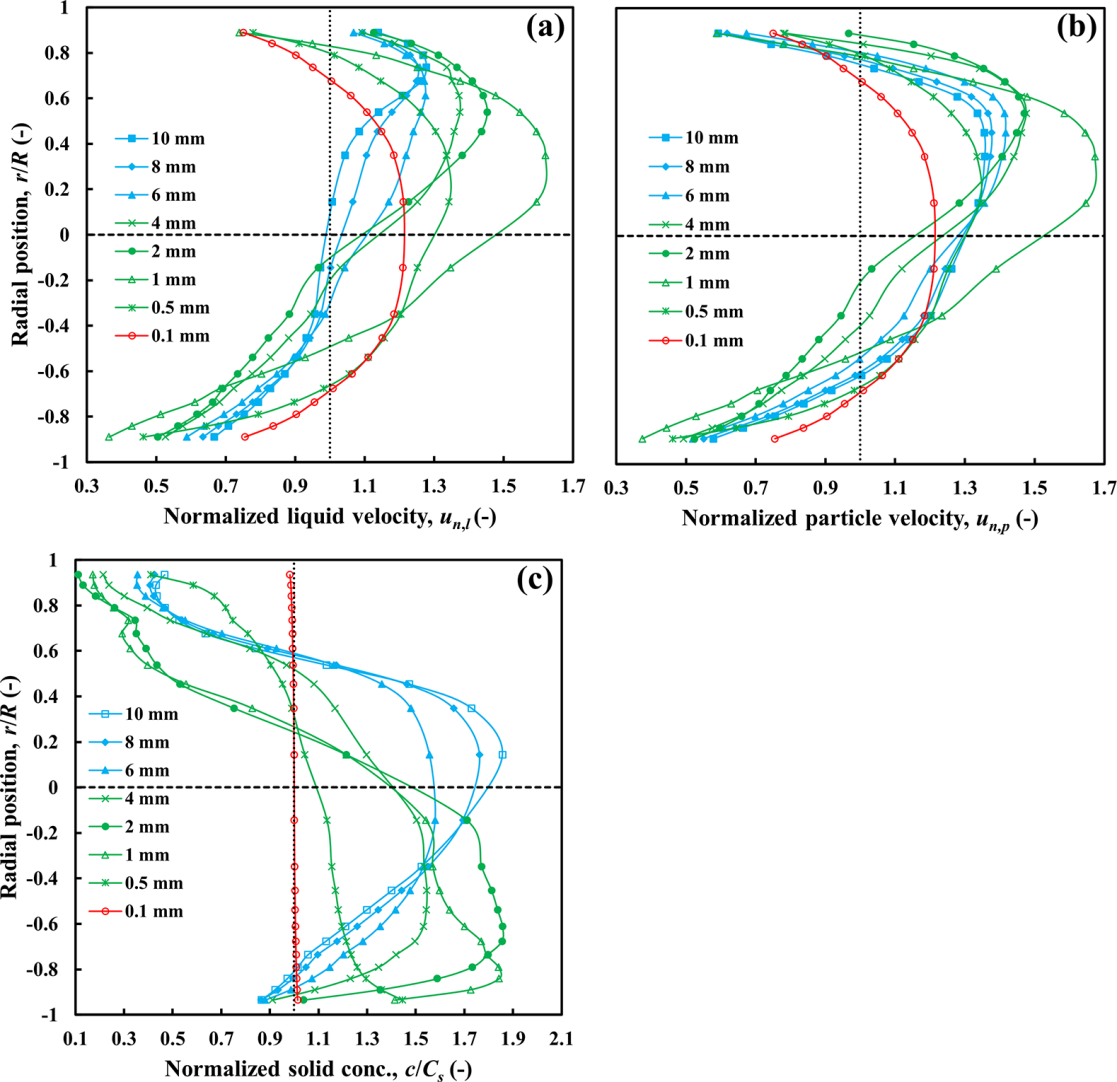
Effects of particle size on radial profiles of (a) liquid velocity, (b) particle velocity, and (c) particle concentration, *C*_*s*_ = 30 vol %, ρ_*r*_ = 1.5.

For the purpose of discussion, particles are classified in three categories based on the similarity of their profile plots: fine, medium, and coarse, whose profile plots are presented, respectively, in red, green, and blue color in Figures 8–10. Note that the boundaries between these classes of particles are not rigid as they may be affected by other factors, especially particle density in this case.

##### 5.2.1.1 Nearly Neutrally Buoyant Particles (ρ_r_ = 1.02)

The effects of particle size on the liquid and particle velocity profiles and solid phase distributions are presented in Figure 8. The liquid and particle velocity distributions of coarse nearly neutrally buoyant particles (*d*_*p*_ = 4–10 mm) lie within a narrow range of each other and are approximately symmetrical about the centerline; going from 4 to 10 mm, the profile becomes slightly flatter, that is, more uniform, with a smaller maximum (Figures 8a,b). These effects are also observed in the particle concentration profiles (Figure 8c). It should be note that in both sets of plots, the rate of decrease of the curve maximum reduces as particle size increases.

Fine nearly neutrally buoyant particles (*d*_*p*_ < 1 mm) behave differently from coarse particles such that the maximum velocity increases as the particle size increases. The particle distribution profiles are also markedly different, as local concentration is almost uniform across the pipe (Figure 8c). This class of particles moves in homogeneous flow. The velocity profiles of medium size particles (1 ≤ *d*_*p*_ ≤ 2 mm) do not exhibit any clear trend. However, increasing *d*_*p*_ from 1 to 2 mm entrains a large change in particle distribution, going from nearly uniform to being very sharp with a large accumulation of particles in the core region of the pipe reaching the maximum packing fraction (0.63) at the center. It seems that particle distribution in this intermediate class is particularly sensitive to particle size.

It also appears from the velocity profiles that nearly neutrally buoyant particles of fine and medium size have a negligible slip velocity since their velocity profiles approximately match the liquid velocity profiles. Thus, the suspension behaves like single phase flow. Coarse particles, however, exhibit a significant slip velocity, moving faster than the liquid in most regions of the pipe except near the wall where they lag the liquid.

##### 5.2.1.2 Slightly Denser Particles (ρ_r_ = 1.09)

Increasing the density ratio to 1.09 introduces significant changes in the two-phase flow behavior, and especially in the velocity profiles, as shown in Figure 9. For coarse particles (*d*_*p*_ ≥ 6 mm), the liquid and particle velocity profiles are asymmetrical about the centerline, with particles and liquid phase moving faster in the upper part of the pipe. Increasing particle size does not affect the particle velocity profiles but causes a significant slowing down of the liquid phase in the core, which is attributed to increased solid accumulation in this region of the pipe, as shown in Figure 9c. Of all three classes, coarse particles are the only ones with a significant slip velocity.

Fine particles (*d*_*p*_ ≤ 1 mm) have liquid and particle velocity profiles that are symmetrical about the pipe axis, with a substantial flat core region that moves as a plug, akin to a viscoplastic fluid with an apparent yield stress. With the slight exception of the 1 mm particles, these particles are also distributed almost uniformly across the pipe section similar to the fine nearly neutrally buoyant particles discussed above (Figure 9c). Particles of 1 mm diameter seem to represent the upper limit of this class, a transition point between the fine and medium size classes, as their radial distribution seems to mark the beginning of a shift from homogeneous flow. The intermediate category (1 < *d*_*p*_ < 6 mm) shows asymmetrical liquid and particle velocity profiles with a maximum above the centerline. The radial distribution of 2 mm particles exhibits some accumulation near the bottom, while most of the 4 mm particles accumulate near the center.

##### 5.2.1.3 Heavy Particles (ρ_r_ = 1.5)

The liquid/particle velocity and solid concentration profiles for particles of density ratio 1.5 are presented in Figure 10. Coarse (*d*_*p*_ ≥ 6 mm) and intermediate (0.5 ≤ *d*_*p*_ < 6 mm) particles produce asymmetric liquid and particle velocity profiles, with a maximum velocity appearing well above the centerline. Again, coarse particles are the only ones exhibiting a significant slip velocity. Medium size particles show substantial accumulation in the bottom part of the pipe, while coarse particles accumulate mostly above the centerline.

Fine particles (*d*_*p*_ < 0.5 mm) generate solid and liquid velocity profiles that are symmetrical about the centerline and are completely uniformly distributed across the pipe section. While increasing particle density reduces the upper size limit of particles belonging to the fine particle category, small enough particles will always move in the homogeneous flow regime with a velocity distribution resembling that of a yield stress fluid.

**Figure 11:**
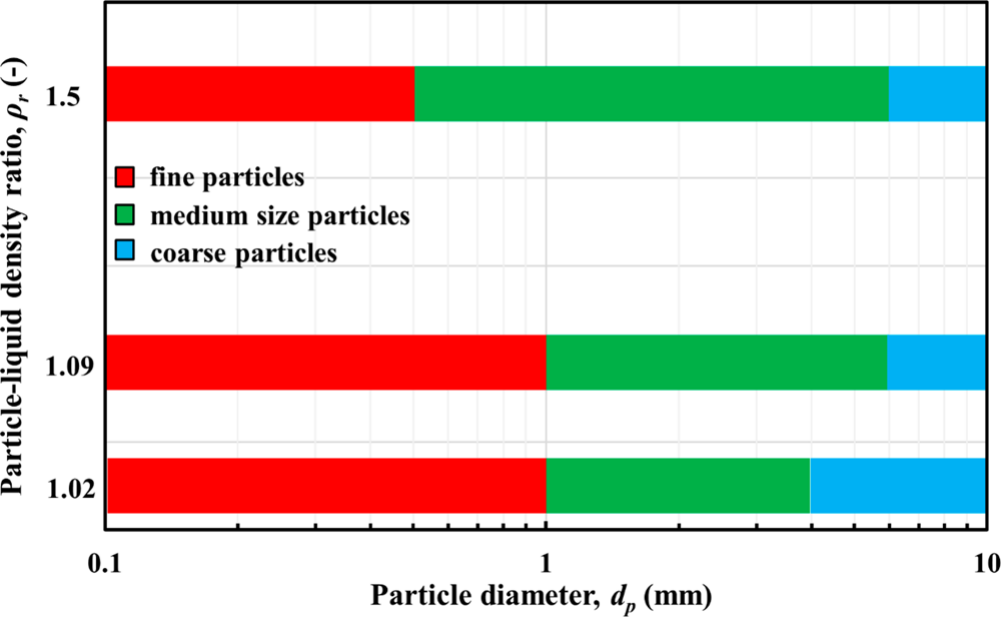
Particle classification as a function of particle size and particle–liquid density ratio.

The above results suggest that particles may be categorized into classes as a function of their size and particle–liquid density ratio, as depicted in Figure 11. As discussed above, the effects of particle size on the two-phase flow in terms of the phase velocity profiles and radial particle distribution are minimal in the case of fine and coarse particles but are significant for the intermediate class. Increasing the particle–liquid density ratio brings about a broadening of the intermediate size class, thus widening the range of particle–liquid flows being affected by particle size. For example, whether a particle is classified as fine depends not only on its size but also its density, that is, the range of particles considered as fine shrinks in terms of size as their density increases and they become heavier, so it is the mass and inertia of the particle that are pertinent.

#### 5.2.2 Force Balance

**Figure 12:**
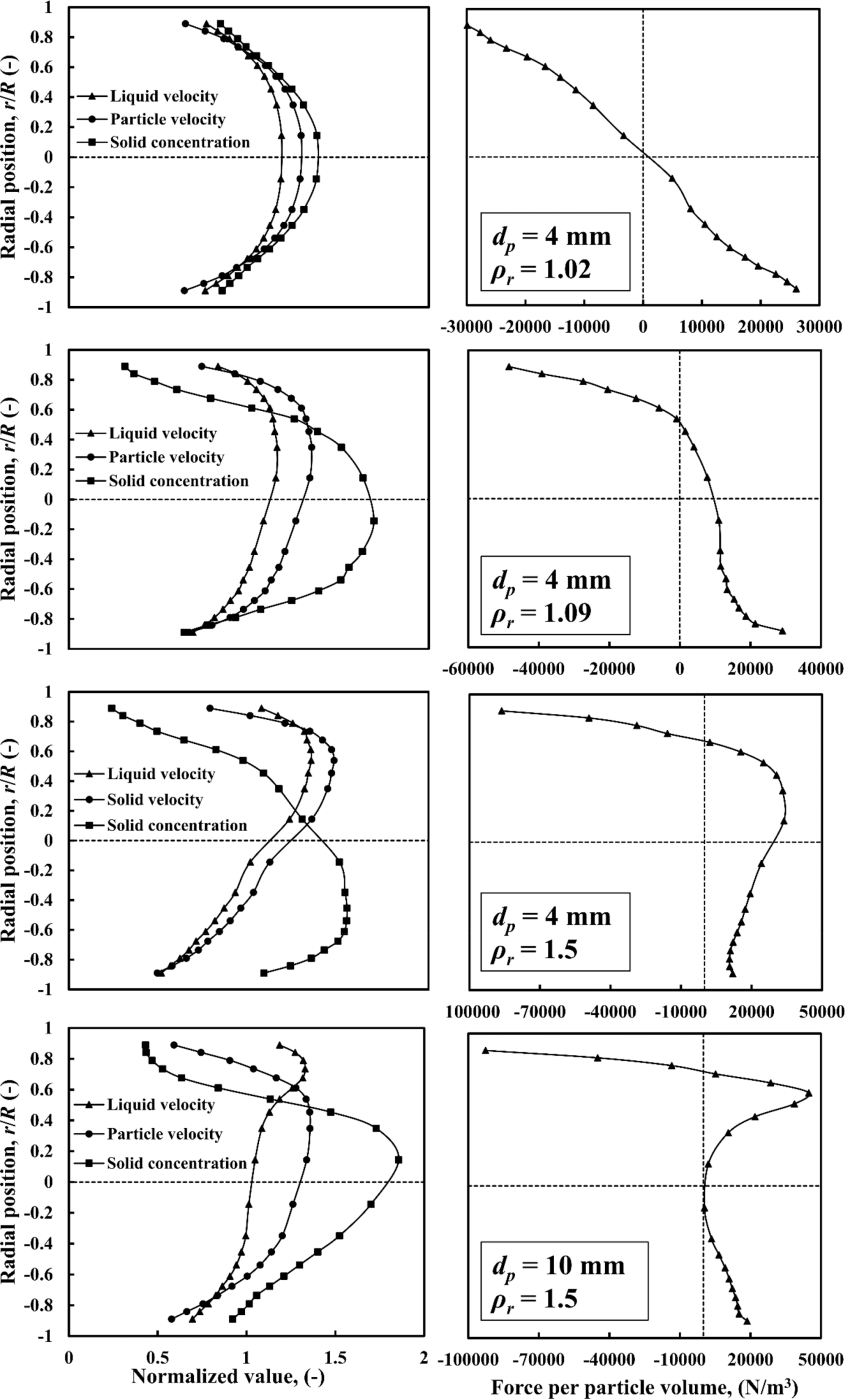
CFD-predicted net force profile as a function of particle size and particle–liquid density ratio: *C*_*s*_ = 30 vol %.

To help interpret the phenomena observed above regarding radial particle distribution, a quantitative analysis of the forces governing the two-phase flow is presented in Figure 12. The normalized profiles of liquid and particle velocity as well as particle concentration are presented for different cases of particle size and particle–liquid density ratio, alongside plots of the net force acting on the particles. The force balance in the vertical direction included the lift, gravity, buoyancy, drag, turbulent dispersion, virtual mass, and pressure gradient forces, per unit particle volume. The predominant forces that influenced particle distribution across the pipe section were the gravity, lift, buoyancy, and pressure gradient forces, other forces being negligible.

For nearly neutrally buoyant particles (*d*_*p*_ = 4 mm, ρ_*r*_ = 1.02), the net force acting is symmetrical about the origin. This causes radial migration of particles in a way that generates an axisymmetrical distribution of solids with a maximum at the center. Increasing ρ_*r*_ to 1.09 and 1.5, the force distribution becomes asymmetric crossing the zero-line well above the centerline. The negative force values in the top part of the pipe cross-section are much greater, which causes enhanced downward particle migration leading to increased accumulation of solids in the lower part of the pipe and, consequently, higher liquid and particle velocities at the top.

For coarse particles with *d*_*p*_ = 10 mm, ρ_*r*_ = 1.5, a positive peak in the net force appears in the upper region of the pipe and less of the pipe cross-section is covered by negative force values, mainly close to the upper wall. Thus, particles in the bottom region experience a net upward force toward the center, whereas particles near the top experience the combination of a large downward negative force and a smaller upward positive force. An equilibrium is, thus, reached whereby a maximum solid fraction is established near the center of the pipe.

## 6 Conclusion

CFD simulations using the Eulerian–Eulerian numerical approach with appropriate KTGF models have been used to predict the flow field of turbulent two-phase particle–liquid flow in a horizontal pipe. The simulations have been fully and successfully validated using experimental measurements acquired by a unique technique of positron emission particle tracking. The numerical approach adopted has been able to predict, with a high degree of accuracy, the radial liquid and particle velocity profiles as well as radial solid phase distribution at solid loadings varying from low to high for suspensions of nearly neutrally buoyant as well as dense particles.

Nearly neutrally buoyant particles exhibit radial velocity profiles for both liquid and particles that are approximately symmetrical about the centerline, while for dense particles, the velocity profiles are asymmetrical, with a maximum located well above the centerline. This degree of asymmetry in such velocity profiles increases further with particle concentration. At low to medium concentrations, both dense particles as well as nearly neutrally buoyant particles show significant accumulation at the bottom of the pipe. The particle distribution profiles, however, are much sharper for dense particles. Particle distribution gradually loses its asymmetry as solid loading increases, becoming almost symmetrical at high concentrations, with a maximum at the center.

Results suggest that particles may be categorized into classes such as fine, medium, and coarse based on the similarity of their phase velocity profiles as well as particle distribution. For a given liquid and flow regime, the borderlines separating the particle classes are determined by size and particle–liquid density ratio. The effects of particle size on the two-phase flow in terms of the phase velocity profiles and radial particle distribution are minimal in the case of fine and coarse particles but are significant for the intermediate class size. Increasing the particle–liquid density ratio broadens the intermediate size class, thus widening the range of particle–liquid flows being affected by particle size. A quantitative analysis of the forces governing the two-phase flow has been presented to help interpret the observed flow phenomena.

## Supporting Information Available

Comparison of CFD-predicted and experimental PEPT profiles of liquid velocity, particle velocity, and particle concentration for other nearly neutrally buoyant particles (PDF)
