# Supplementary material for: Numerical and Experimental Investigations of Horizontal Turbulent Particle–Liquid Pipe Flow
Source: Ind Eng Chem Res. 2022 Aug 4;61(32):12040–51. doi: 10.1021/acs.iecr.2c02183 (PMC9389582; doi:10.1021/acs.iecr.2c02183)
Supplement: Supplementary file 1 — ie2c02183_si_001.pdf [file ie2c02183_si_001.pdf]

Supporting information for

Numerical and Experimental Investigations of Horizontal Turbulent Particle-Liquid Pipe Flow

ZhuangJian Yang, Chiya Savari, Mostafa Barigou*

School of Chemical Engineering, University of Birmingham, Edgbaston, Birmingham B15 2TT, UK

*Corresponding author: m.barigou@bham.ac.uk

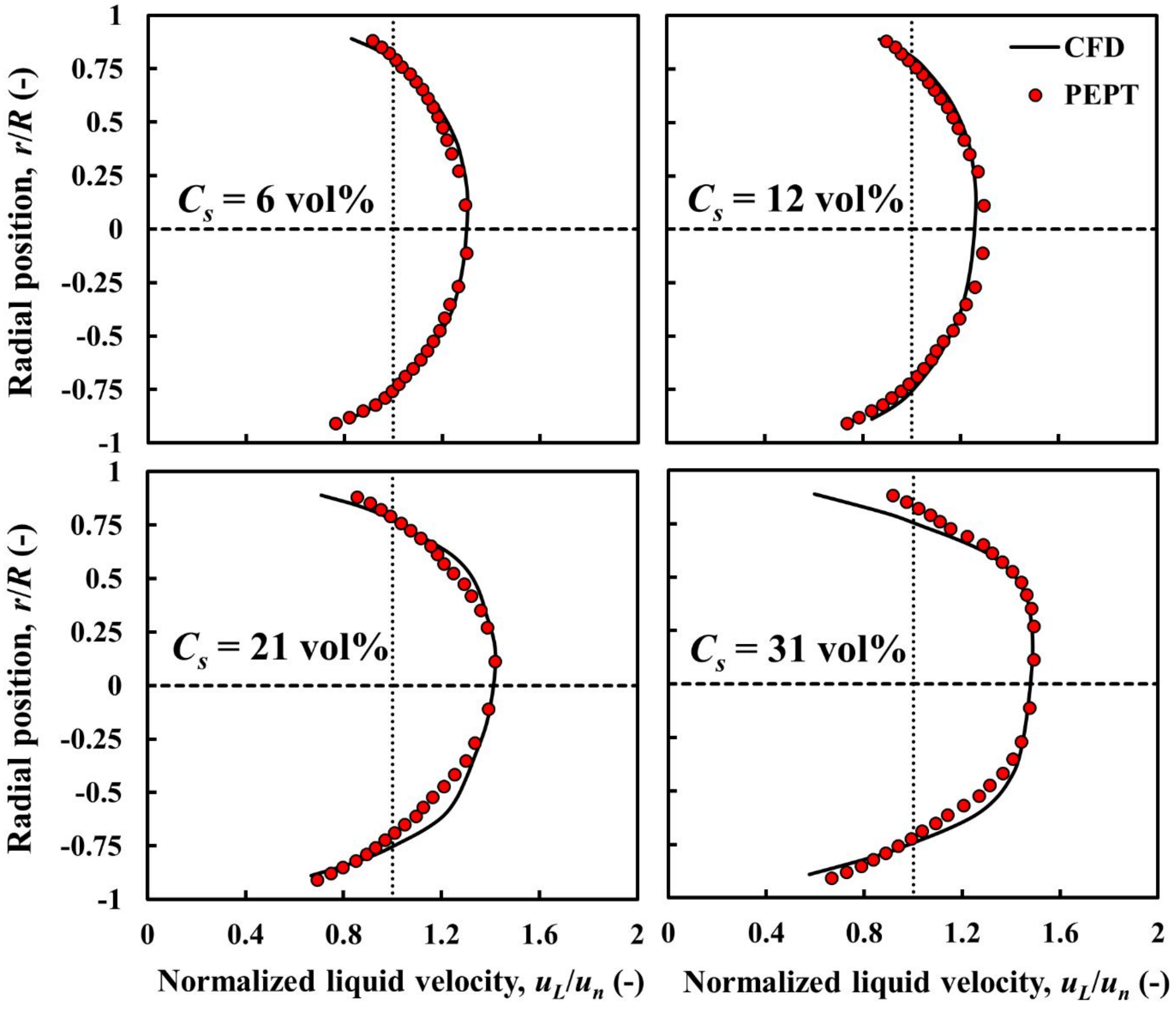

Figure S1. CFD-predicted and experimental PEPT liquid velocity profiles compared: nearly-neutrally buoyant particles, ρ_r = 1.02; d_p = 2 mm.

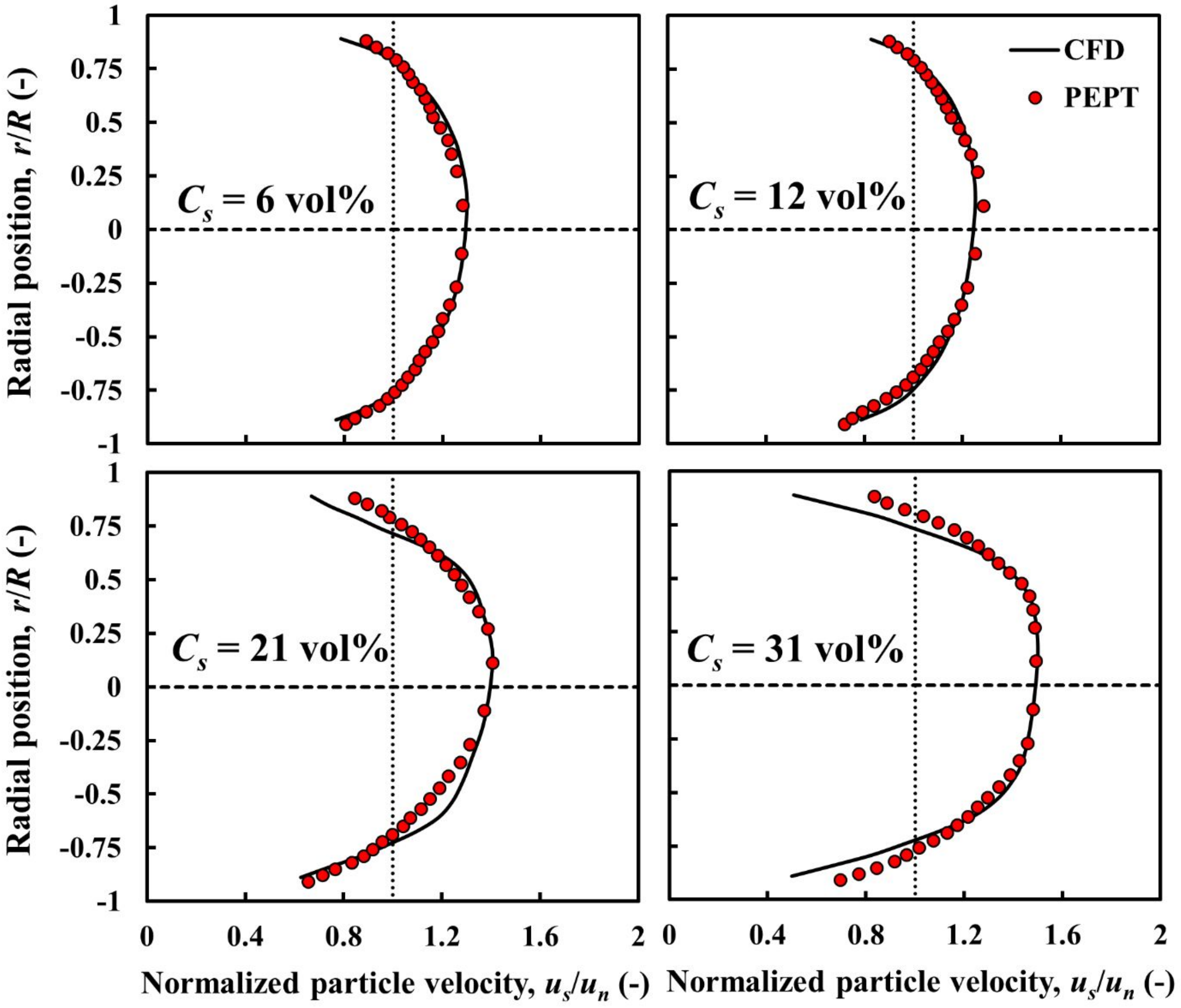

Figure S2. CFD-predicted and experimental PEPT particle velocity profiles compared: nearly-neutrally buoyant particles, ρ_r = 1.02; d_p = 2 mm.

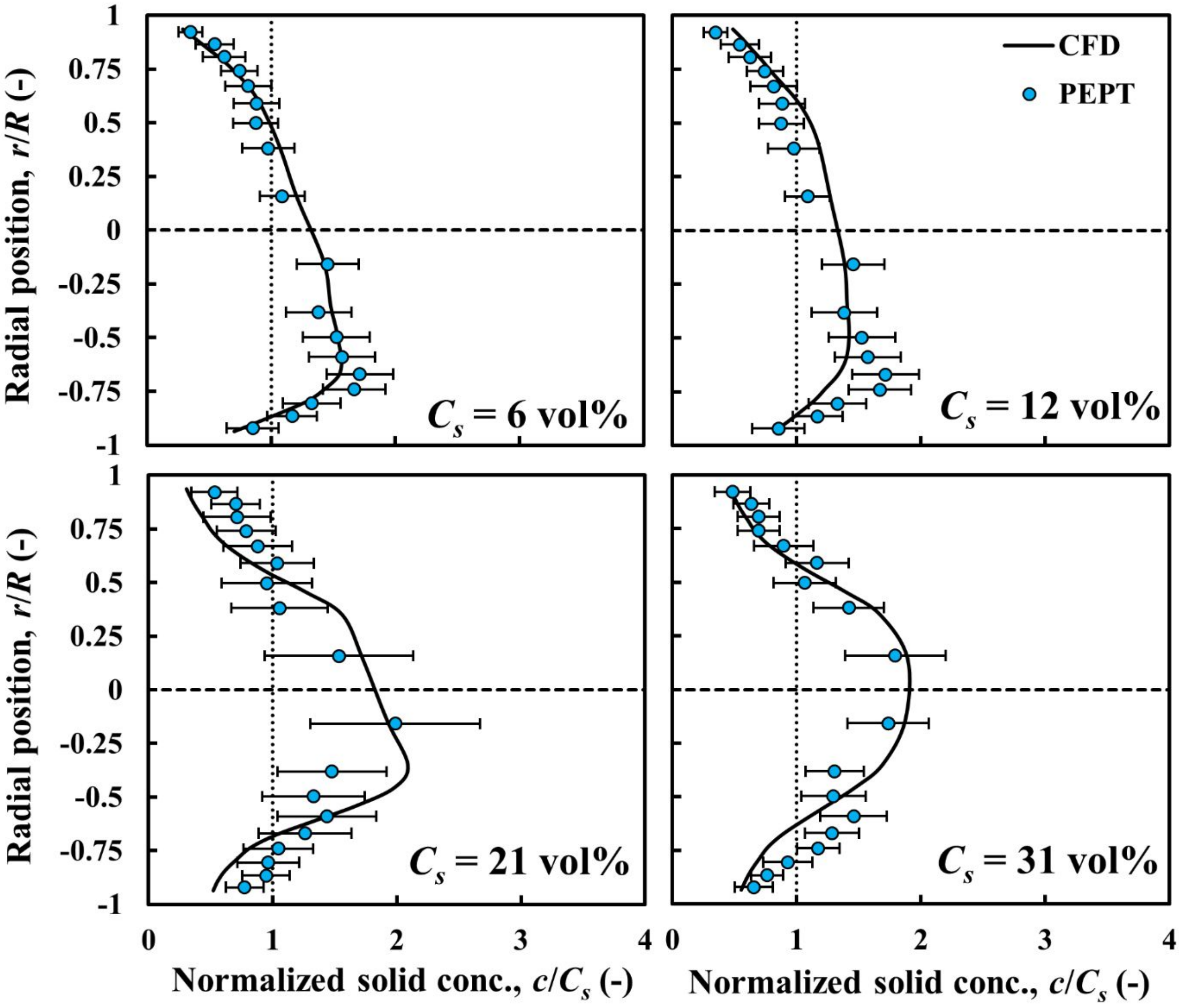

Figure S3. CFD-predicted and experimental PEPT particle concentration profiles compared: nearly-neutrally buoyant particles, ρ_r = 1.02; d_p = 2 mm.
